# THE WOMEN'S HEALTH INITIATIVE (WHI): STILL LEARNING FROM 161,808 POSTMENOPAUSAL WOMEN

**DOI:** 10.1093/geroni/igz038.1288

**Published:** 2019-11-08

**Authors:** Andrea Z LaCroix, Andrea Z LaCroix

**Affiliations:** 1 University of California San Diego, La Jolla, California, United States; 2 Family Medicine and Public Health, University of California, San Diego, La Jolla, California, United States

## Abstract

The WHI enrolled 161,808 women ages 50-79 into 1-3 Clinical Trials (n=68,132) or the Observational Study (n=93,676) from 1993-1998. As of March 31, 2018, 70,812 women are alive and continue to be enrolled including 10,179 over the age of 90, 4588 Black/African American, and 1998 Hispanic/Latina women. 54,877 women have died, including 17,010 from cardiovascular disease and 14,553 from cancer. 64,344 reported a fracture during follow-up. Between 2012-2014, 7875 women completed a brief in-person visit as part of The Long Life Study. This examination included the Short Physical Performance Battery, grip strength, height, weight, blood pressure, heart rate and a blood draw. Genetic data of various types have been collected for over 30,000 WHI participants. Whole genome sequencing data is available for over 11,000 WHI participants. Over 1700 manuscripts have been published. The WHI biospecimen repository contains millions of stored biospecimens. CMS (Medicare) data is available and regularly updated.

